# Prevalence, timing and characteristics of delirium preceding dementia with Lewy bodies: a retrospective case series

**DOI:** 10.1007/s00415-025-13193-y

**Published:** 2025-06-21

**Authors:** Janice Taylor, Chaminda Gunawardana, Simon J. G. Lewis, Elie Matar

**Affiliations:** 1 Faculty of Medicine, Parkinson’s Disease Research Clinic, Macquarie University, 75 Talavera Road, Macquarie Park, NSW 2113 Australia; 2 Faculty of Medicine, Macquarie University, Macquarie Park, NSW Australia; 3 Faculty of Medicine and Health, Central Clinical School, University of Sydney, Camperdown, NSW Australia; 4 Department of Neurology, Royal Prince Alfred Hospital, Camperdown, NSW Australia; 5 Sydney Medical School, Faculty of Medicine and Health, University of Sydney, Camperdown, Australia; 6 Faculty of Medicine and Health, Brain and Mind Centre, University of Sydney, Camperdown, NSW Australia; 7 Centre for Integrated Research and Understanding of Sleep (CIRUS), Woolcock Institute of Medical Research, Sydney, NSW Australia

**Keywords:** Delirium, Dementia with Lewy bodies, Prodromal Lewy body dementia, Mild cognitive impairment

## Abstract

**Background:**

Consensus diagnostic criteria for delirium-onset dementia with Lewy bodies are lacking. This retrospective study aimed to identify delirium occurring in the prodrome of dementia with Lewy bodies (DLB), exploring delirium-onset DLB as an entity and its natural history.

**Methods:**

Thirty-four participants with an established diagnosis of probable DLB from an outpatient neurology clinic in Sydney underwent a structured telephone interview to identify episodes of delirium. The timing, precipitants, and phenomenology of each episode were documented. Core and supportive features from the proposed diagnostic criteria for DLB were evaluated.

**Results:**

26% of the participants experienced delirium prior to diagnosis of DLB, with one participant experiencing multiple episodes in the 24 months before diagnosis. Of these cases, 66% demonstrated some core and supportive features of DLB at the time of their delirium. In addition to expected triggers (e.g. surgery), international (long-haul) air flight was identified as one of the commonest precipitants for delirium in this group. Those DLB cases who fulfilled the proposed research criteria for a delirium-onset prodrome experienced a shorter time from symptom onset to dementia than those DLB patients with no history of pre-diagnosis delirium (26 vs 40 months).

**Conclusions:**

These findings support delirium as a marker of prodromal DLB and suggest delirium-onset cases exhibit a more rapid progression to dementia, offering a focus for future studies. Identifying delirium as a potential early feature of DLB could aid in earlier recognition and improve diagnostic accuracy, particularly in individuals presenting with precipitants such as international air travel.

**Supplementary Information:**

The online version contains supplementary material available at 10.1007/s00415-025-13193-y.

## Introduction

Dementia with Lewy bodies (DLB) is the second most common neurodegenerative dementia after Alzheimer’s dementia (AD), accounting for 4.2% of dementia diagnoses in community-dwelling populations [1]. The combined prevalence, including neuropathological studies, is higher at around 15% [2, 3], reflecting high rates of misdiagnosis due to the variable cognitive, neuropsychiatric and motor presentations associated with heterogenous Lewy body pathology distributions [4], and varying AD-related co-pathology [5–9]. Current criteria for a clinical diagnosis of probable DLB requires the presence of dementia in association with two or more core clinical features, including spontaneous visual hallucinations, cognitive fluctuations, parkinsonism and rapid eye movement sleep behaviour disorder (RBD), with or without indicative biomarkers, or the presence of one core clinical feature with one or more indicative biomarkers [10].

Patients diagnosed with DLB have a median survival of 4.1 years from diagnosis, with 95% mortality at 10 years despite their first clinical symptoms and signs manifesting up to 15 years or more before diagnosis [11, 12]. This means that clinicians, researchers, and, most importantly, patients are likely to be missing the opportunity for disease modification and disease-specific management strategies to reduce morbidity and mortality at the earliest point in time where they might be most effective. To address this shortfall, three prodromal phenotypes were proposed [11]: mild cognitive impairment with Lewy bodies (MCI-LB), psychiatric-onset DLB and delirium-onset DLB. Whilst detailed research criteria for the diagnosis of possible and probable MCI-LB were outlined, there was insufficient evidence to formulate criteria for psychiatric-onset and delirium-onset DLB. Psychiatric-onset DLB should be suspected in patients presenting with late-onset major depressive disorder, late-onset psychosis or very late-onset psychosis of severity that may require hospitalisation [11]. One recent case series suggests female predominance with average age of psychiatric symptom onset greater than 60 years and depression as the most common presenting feature [13, 14]. Whilst it was concluded that there was insufficient evidence to support formal diagnostic criteria for prodromal delirium-onset DLB, McKeith et al. [11] suggest it should be suspected in patients presenting with delirium episodes with inadequate provoking factors, prolonged or recurrent deliriums, or those who later develop progressive cognitive decline or subsequent dementia.

Delirium is characterised by an acute fluctuating disturbance in levels of attention and awareness associated with a change in cognition that cannot be otherwise explained by an evolving neurocognitive disorder [15]. In contrast, dementia represents a clinical diagnosis evidenced by a chronic progressive decline across two or more cognitive domains that results in a loss of independence in everyday activities [15]. However, there is emerging evidence supporting a bidirectional relationship between delirium and dementia, with neurodegeneration predisposing to delirium, and delirium itself resulting in neuronal damage leading to dementia [16–18].

Delirium episodes are believed to occur in the prodromal period and throughout the clinical course of many neurodegenerative diseases, but especially DLB [19]. Indeed, in the year before diagnosis, patients diagnosed with DLB have a 6 times greater incidence of delirium compared to those who develop Alzheimer’s dementia [19]. The duration of delirium is independently associated with worse global cognition and executive function [20, 21]. Prior studies suggest between 6 and 50% of patients diagnosed with DLB will experience an episode of delirium in the prodromal period, with a median onset at 11 months pre-dementia diagnosis ranging between 1 and 66 months [14, 19, 22–25]. In addition, a quarter of these patients experience multiple episodes of delirium prior to diagnosis [24]. Importantly, DLB patients are at increased risk due to antipsychotic use as first-line pharmacological management of delirium, increasing the mortality risk [10]. Clearly, accompanying core clinical features of DLB are likely to be difficult to discern due to the overlap of the symptomatology of delirium with spontaneous visual hallucinations and cognitive fluctuations [26], as well as antipsychotic and antidepressant effects on parkinsonism and RBD, respectively [11, 14].

Delirium occurs in 23% of hospitalised patients, with an incidence of up to 70% of ICU admissions [27]. Despite the common occurrence of delirium, there are few studies appraising the inciting event and phenomenology of delirium episodes occurring in the prodromal period of DLB [24, 28–30]. This is especially important to help clinicians decide which patients require ongoing monitoring for the development of DLB following an acute episode of delirium. Therefore, the current study aimed to identify and characterise the syndrome of delirium occurring in the prodromal period of DLB. Clinical assessment, medical records and informant accounts were used to explore the prevalence, triggers and features of delirium as well as the presence of associated core and suggestive features in the prodromal period and at the time of diagnosis. The findings were synthesised to develop a set of heuristics to help prompt clinicians to consider a diagnosis of prodromal DLB following an episode of delirium.

## Methods

In this study, 57 patients with a diagnosis of probable DLB, as per the current consensus criteria [10], were retrospectively identified from a cohort of community-dwelling patients attending a specialist Parkinson’s disease and related disorders neurology clinic in Sydney, between January 2017 and July 2024. The primary source of referrals was from general practitioners (*n* = 33), neurologists (*n* = 10) and geriatricians (*n* = 6). The remaining referrals were from other specialists (*n* = 6) and psychiatrists (*n* = 2). Patients and their caregivers were invited via email or telephone call to participate in a semi-structured telephone questionnaire exploring symptomatology prior to the diagnosis of DLB. A future appointment to conduct the interview was arranged to allow caregivers time for the improved recall of events and to gather supportive medical documentation.

The semi-structured telephone questionnaire was developed by JT and reviewed by all the authors to ensure appropriate language and content. Questions were designed to identify episodes of delirium prior to diagnosis based upon the 5th edition of the Diagnostic and Statistical Manual diagnostic criteria for delirium [15]. A delirium episode was defined by an acute change in mental status as noted by the caregiver associated with confusion, disorientation, disturbance in speech, day/night disorientation, behavioural disturbance, or reduced level of consciousness that may have been associated with psychotic features of hallucinations across any sensory modality, or delusions. The telephone script included triggering phrases to assist caregiver identification of observed behaviours reflective of the DSM criteria for delirium, including: “a sudden change in behaviour or demeanour”, “problems following an anaesthetic/surgery, infection, or medication introduction/change?”, “muddled or disoriented to the time of day”, “awake and confused at unusual times/overnight”, “seeing or hearing things that weren’t present”, “paranoid or suspicious thoughts”, and/or “excessively sleepy/drowsy during the day/difficulty rousing them”. To increase sensitivity, any positive response indicating acute change in mental status was considered suggestive of delirium and the associated trigger, phenomenology and duration of each delirium episode were recorded. Duration of delirium was determined by caregiver recall of time to resolution of symptoms with the options of “*days*”, “ < *2 weeks*”, “*2 weeks to one month*”, *or *“ > *one month*”*.* Further questions aimed to identify the first core clinical feature or suggestive prodromal subtype as noted by the caregiver prior to diagnosis, alongside core and supportive clinical features at diagnosis and time of onset pre-diagnosis. Core clinical features included spontaneous visual hallucinations, cognitive fluctuations, parkinsonism and rapid eye movement sleep disorder. Supportive clinical features at diagnosis included anxiety, depression, apathy, auditory hallucinations, tactile hallucinations, delusions, anosmia, constipation, urinary incontinence, falls, orthostatic hypotension and neuroleptic sensitivity [10]. Suggestive prodromal subtypes were identified if the first notable symptom was of cognitive change for MCI-LB, delirium for delirium-onset DLB, and first episode psychosis or depression for psychiatric-onset DLB [11].

Telephone interviews were performed by a clinician with experience in identification and diagnosis of delirium, as well as the clinical and supportive features of DLB. This was supplemented with, and cross-referenced against, information contained within the initial Neurologist clinic consultation letter as to reduce the risk of recall bias and missing data. If differences in the timing or symptomatology were identified, the information contained within the consultation letter was considered most accurate given the shorter duration between symptom development and recall of information, with the exception of delirium episodes, where the caregiver report took precedence. Presence of cognitive fluctuations was gathered from the neurologists first clinic letter rather than from caregiver history, to maximise sensitivity of capturing caregiver identification of possible hypoactive delirium episodes. File review was performed to obtain results of Montreal Cognitive Assessment (MoCA) scores within 6 months of diagnosis. Data were collated and deidentified by JT.

Group-level analyses were conducted using Mann–Whitney *U* tests for continuous variables (age and MoCA scores), and Chi-squared tests to assess differences in categorical variables, including sex and the presence of core and supportive diagnostic features at the time of DLB diagnosis. All participants gave informed consent for their involvement in this study (HREC 2103/945).

## Results

### Demographics

Of the 57 patients identified, 34 participants and caregiver dyads agreed to participate in the semi-structured telephone interview. Of these, 26% (*n* = 9) experienced an episode of delirium prior to diagnosis, with one participant experiencing three episodes within the 24 months preceding their diagnosis of DLB. The average age at DLB diagnosis in the delirium group was 73 years compared to 76 years in the no-delirium group, with males predominating each group (see Table 1). Of the 23 participant and caregiver dyads who did not proceed with participation, 16 were not contactable despite multiple attempts via telephone or email. Of the remaining seven, one was subsequently excluded after review due to not meeting clinical criteria for DLB, three declined participation due to bereavement following death of the participant with DLB, and the remaining three offered no reason. Demographic details of non-participants are also provided in Table 1.

**Table 1:** Participant demographics

Variable	% (*n*)/median (range)				
	Non-participants	Participants			
	Non-participant cohort (*n *= 23)	Participant cohort (*n* = 34)	Delirium 26% (*n* = 9)	No delirium 74% (*n* = 25)	*p*-value
Age at diagnosis	76 (62–88)	75 (62–84)	73 (69–80)	76 (62–84)	0.938
Sex (% male)	81% (17)	77% (26)	78% (7)	76% (19)	0.914
		(*n* = 21)	(*n* = 7)	(*n* = 14)	
MoCA	17 (7–26)	20 (6–26)	22 (17–26)	19 (6–23)	0.114

*MOCA* Montreal Cognitive Assessment Score

### Timing and duration of delirium relative to DLB diagnosis

The average age at time of first delirium episode was 70 years, ranging between 61 and 80 years. The average time from delirium to diagnosis was 48 months, with a median time of 24 months. The longest duration between delirium episode and diagnosis was 12 years for one participant who experienced delirium associated with opioid use post a total knee replacement.

### Precipitants of delirium

Delirium precipitants included circadian rhythm dysregulation associated with international air travel (*n* = 3), anaesthesia/surgery (*n* = 3), infection/inflammation (*n* = 3), pain in the setting of delayed diagnosis of hip fracture (*n* = 1) and first opioid exposure (*n* = 1). This includes data for one participant who experienced three delirium episodes prior to their diagnosis of DLB. Where participants experienced multiple precipitants for a single delirium episode (i.e. fracture, pain, surgery, opioid exposure and circadian dysregulation), efforts were made to group and identify the most prominent precipitants for each delirium event. Surgical procedures included hernia repair, transurethral resection of the prostate, spinal surgery and cholecystectomy for gangrenous gallbladder. Infective and inflammatory responses included respiratory tract infection, pneumococcal vaccination and cholecystitis.

### Delirium characteristics

Delirium phenomenology included formed visual hallucinations, paranoid delusions, language and speech disturbance, auditory hallucinations associated with visual hallucinations, and psychomotor agitation (see Table 2). The most frequently observed delirium phenomena were that of visual hallucinations and paranoid delusions in 73% and 45% of participants, respectively. Half of the participants were hospitalised during the episode of delirium, with one episode not recognised by the attending healthcare professionals. Delirium duration ranged from days to weeks, with 36% experiencing fluctuating delirium persisting for greater than 1 month.

**Table 2:** Characteristics and phenomenology of delirium episodes

Delirium characteristics	
Average time from delirium to diagnosis	48 months
Median time from delirium to diagnosis (range)	24 months (3–144)
Precipitating event	
Circadian rhythm dysregulation/flight	27% (3)
Anaesthesia/surgery	27% (3)
Infection/inflammatory response	27% (3)
Opioid use	9% (1)
Pain	9% (1)
None	0% (0)
Delirium phenomenology	
Visual hallucinations	73% (8)
Disturbance in attention and arousal	64% (7)
Paranoid delusions	45% (5)
Confused conversation	18% (2)
Auditory hallucinations	9% (1)
Psychomotor agitation	9% (1)
Delirium duration	
Days	36% (4)
2 weeks	27% (3)
≥ 1 month	36% (4)

### Relative appearance of core features

The first identified diagnostic features of DLB (see Supplementary Table 1), as noted by caregivers in both the delirium and no-delirium groups, were cognitive change (53%) followed by parkinsonism (32%). Caregiver recognition of significant cognitive change occurred between 12 and 72 months, with an average onset of 24 months pre-diagnosis. Emergence of the first core features occurred most commonly within 2 years of diagnosis, with RBD and parkinsonism presenting up to 10 and 5 years prior, respectively. Two caregivers noted the delirium episode as the first notable symptom heralding a DLB diagnosis. One had associated features of RBD, visual hallucinations (preceding the delirium episode), apathy and orthostatic hypotension preceding the delirium event, with the other having RBD alone. Although not meeting statistical significance, the average time of first DLB core symptom onset was 26 months in the delirium group compared to 40 months in the no-delirium group, suggesting a shorter prodromal period for these patients. Of those experiencing delirium, four participants had core features present at the time of delirium, three of which were RBD (see Table 3). The most common core features at diagnosis were RBD (88%), bradykinesia (85%) and cognitive fluctuations (59%). There was no significant difference in frequency of each of the core features at diagnosis between the delirium and no-delirium groups (see Table 4).

**Table 3:** Details of delirium episodes by case

Gender	Age at diagnosis	Age at time of delirium	Timing of delirium pre-diagnosis	Clinical features pre-delirium	Delirium precipitant	Characteristics of delirium
F	73	61	144	None	Opioids	Visual hallucinations
M	69	65	48	RBD (84)	Anaesthesia/surgery—hernia repair	Disorientation Fluctuating (reduced) level of consciousness
M	70	69	6	Apathy/anxiety (60)	Anaesthesia/surgery—transurethral resection of prostate	Disorientation
						Visual hallucinations
				Tremor (36)		Paranoid delusions
M	80	80	3	OH (72)	Pain—conservatively managed hip fracture	Fluctuating confusion/disorientation
				RBD/apathy (24)		Formed visual hallucinations—children and adults
						Auditory hallucinations—adult visual hallucinations threatening him
				VH (18)		Paranoid delusions—based upon visual hallucinations
						Delusions—trapped in video game needing to achieve a target each night
M	77	75	24	RBD (84)	Anaesthesia/surgery—spinal surgery	Visual hallucinations—scenes of “UFOs and cheesecakes” around the room
						Paranoid delusions—believes “nursing staff hosting cocktail parties overnight”
						Fluctuating confusion
		76	12		Vaccination—pneumococcal	Visual hallucinations—seeing people within the home
		77	7		Infection—respiratory	Visual hallucinations—seeing people within the home
M	70	66	48	None	Flight	Fluctuating confusion/disorientation Confused conversation
M	77	68	108	None	Infection/anaesthesia/surgery—gangrenous gallbladder	Visual hallucinations
						Fluctuating confusion/disorientation
						Delusions—needing to fix hospital air conditioner with other patients
						Paranoid delusions
						Psychomotor agitation
M	80	78	24	Anosmia (360)	Flight	Fluctuating confusion/disorientation
						Confused conversation
						Executive dysfunction—unable to use seatbelt or toilet on plane
F	70	68	24	SCI (24)	Flight	Visual hallucinations—animals
						Paranoid delusions—people being arrested by police on the flight
						Fluctuating confusion/disorientation

Characteristics and timing of each delirium episode, including presence and timing (months) of core and supportive features prior to diagnosis

*RBD* rapid eye movement sleep behaviour disorder, *OH* orthostatic hypotension, *VH* visual hallucinations, *SCI/MCI* subjective cognitive impairment

**Table 4:** Core and supportive features at diagnosis and timing of onset pre-diagnosis

	% (*n*)/Onset (months)									Chi squared test for Independence	
	Overall (*n* = 34)			Delirium-onset DLB (*n* = 9)			No delirium at onset (*n* = 25)				
	Incidence	Onset	Range	Incidence	Onset	Range	Incidence	Onset	Range	*χ*^2^	*p*-value
Visual hallucinations	53% (18)	11	1–36	56% (5)	6	6–18	52% (13)	12	1–36	0.033	0.855
Cognitive fluctuations	59% (20)	3	1–48	56% (5)	1	1–48	60% (15)	3	1–36	0.054	0.816
Tremor	53% (18)	15	3–60	56% (5)	12	3–36	52% (13)	18	3–60	0.034	0.855
Bradykinesia	85% (29)	18	1–60	67% (6)	18	3–48	92% (23)	18	1–60	3.386	0.066
Rigidity	47% (16)	12	3–60	44% (4)	15	3–48	48% (12)	12	3–60	0.034	0.855
RBD	88% (30)	24	1–240	89% (8)	15	1–84	88% (22)	24	1–240	0.005	0.943
SCI/MCI	71% (24)	24	12–72	67% (6)	21	12–48	72% (18)	30	12–72	0.091	0.763
Anxiety	32% (11)	18	1–360	33% (3)	1	1–60	32% (8)	21	12–360	0.005	0.942
Depression	24% (8)	12	1–60	44% (4)	1	1–12	16% (4)	24	12–60	2.976	0.085
Apathy	15% (5)	60	24–84	33% (3)	48	24–60	8% (2)	84	–	0.654	0.419
Auditory hallucinations	3% (1)	6	–	0% (0)	–	–	4% (1)	6	–	0.371	0.543
Tactile hallucinations	6% (2)	27	6–48	0% (0)	–	–	8% (2)	27	6–48	0.765	0.382
Delusions	26% (9)	6	3–36	11% (1)	3	–	32% (8)	9	3–36	1.484	0.223
Delirium	26% (9)	24	3–144	100% (9)	24	3–144	–	–	–	34.000	< 0.001
Anosmia	50% (17)	90	1–360	44% (4)	78	24–360	52% (13)	210	1–360	0.151	0.698
Constipation	56% (19)	1	1–120	67% (6)	1	-	52% (13)	1	1–120	0.577	0.447
Urinary incontinence	21% (7)	6	1–18	22% (2)	10	1–18	20% (5)	6	1–12	0.020	0.888
Falls	21% (7)	6	1–204	22% (2)	5	3–6	20% (5)	12	1–204	0.020	0.888
Orthostatic hypotension	12% (4)	42	6–72	22% (2)	39	6–72	8% (2)	42	12–72	1.290	0.256
Neuroleptic sensitivity	0% (0)	–	–	0% (0)	–	–	0% (0)	–	–	–	–

Incidence displayed as percentage and number. Onset provided as median number of months prior to diagnosis

*MCI* mild cognitive impairment, *RBD* rapid eye movement sleep behaviour disorder, *SCI* subjective cognitive impairment

### Relative appearance of supportive features

The most common supportive features at diagnosis were constipation (56%), anosmia (50%) and anxiety (32%). The earliest pre-diagnostic clinical features noted in both delirium and no-delirium groups were anosmia, apathy and orthostatic hypotension, with first onset at 30, 7, and 6 years and a median onset of 90, 60 and 42 months, respectively. Two participants from the delirium group had one or more supportive features in addition to a core feature at the time of delirium (see Table 3). Two had a single supportive feature and three had no core nor supportive features identified at the time of their delirium. Although not reaching statistical significance, psychiatric symptom onset was closer to the time of diagnosis in the delirium group compared to the no-delirium group, with an average onset of 1–48 months in the delirium group and 6–84 months in the no-delirium group. The most common psychiatric symptoms in the delirium group were depression (44%), anxiety and apathy (33%), in contrast to isolated anxiety (33%) and delusions (33%) in the no-delirium group. One caregiver in the no-delirium group noted onset of paranoid delusions as the first notable symptom 3 years prior to diagnosis. One participant in the delirium group had psychiatric symptom onset prior to their delirium episode with a diagnosis of anxiety and apathy 60 months beforehand. No significant difference was seen in the presence of supportive features at diagnosis between delirium and no-delirium groups (see Table 4).

## Discussion

Delirium is an under-reported and poorly understood prodromal feature of DLB. In this detailed retrospective case series, we sought to systematically document, quantify and characterise delirium episodes preceding the diagnosis of DLB. In line with estimates from previous studies [14, 19, 22–25], we found that 26% of patients with DLB experienced an episode of delirium prior to their diagnosis with one participant experiencing multiple delirium episodes in the 2 years prior to diagnosis. Previous work suggests that the time for phenoconversion to dementia from MCI-LB is between 14 and 38 months [22] and 9 years for psychiatric-onset DLB [14]. Delirium episodes occurred up to 12 years prior to a diagnosis of DLB, with an average of 4 years prior to diagnosis and most commonly occurring around 2 years prior to a DLB diagnosis.

Outside of case reports of DLB presenting with acute cognitive decline following a delirium episode, only one cohort study by Vardy et al. (2013) has examined the time from any delirium episode to the diagnosis of DLB. Their study, based on a review of documentation from the initial assessment, found a median time from the last episode of delirium to DLB diagnosis of 11 months (range 1 month to 5.5 years), with five of 21 participants experiencing multiple delirium episodes pre-diagnosis. The comparably longer duration between onset and diagnosis seen in this study could reflect differences in case ascertainment, with our cohort being 3 years older at diagnosis compared to the published mean age of their cohort (71 years). Findings from our sample, and Vardy et al. (2013), suggest delirium episodes in the prodromal period of DLB were mostly recalled to occur within 5 years of diagnosis and can recur within 24 months of diagnosis. However, we also found delirium can occur up to 12 years prior to diagnosis, suggesting a variable prodromal period. Future prospective cohort studies are needed to determine the true incidence and timing of DLB phenoconversion after an episode of delirium. Similarly, larger prospective studies analysing cognitive performance prior to phenoconversion may glean insights into the cognitive profile of delirium-onset DLB.

Evidence shows morbidity and mortality increase after an episode of delirium [20, 21, 31–33], and previous studies have proposed an interrelationship between delirium and dementia and whether the delirium is a result of, or accelerates, the underlying Lewy body pathology [17]. In our sample, we observed a more rapid progression to dementia in the delirium-onset group, with an average onset of symptoms within 26 months, compared to 40 months in those who did not experience delirium prior to diagnosis. This interrelationship was also observed by Green et al. [34] with their Parkinson’s disease and atypical parkinsonism groups both having increased hazard ratio of developing dementia after an episode of hospitalised delirium [34]. Larger prospective studies combining in vivo biomarkers with neuropathological confirmation are required to better understand this relationship and determine any significance. In the clinical setting, opportunities could arise to reduce the incidence of delirium with the potential for disease modification [35, 36].

Precipitants of delirium in this study were broadly consistent with themes identified in prior studies and case reports [24, 28–30]. Notably, we identified international air flights as one of the most common triggers for delirium and speculate that this is secondary to circadian disruption associated with long-haul flights common to Australians [37]. Interestingly, whilst flights were similarly identified as a potential trigger in DLB in a previous study [24], no such precipitant was identified in a small comparator group of AD patients, possibly suggesting an increased vulnerability to this precipitant in DLB. One hypothesis for this difference could relate to disease-specific pathology within the sleep wake circuitry that proposed to link cognitive fluctuations and circadian dysfunction in patients with DLB [38–40], with longer duration cognitive fluctuations and delirium in DLB potentially reflecting overlapping pathophysiological processes [38]. Clinically, the entities are difficult to differentiate [11, 26], and previous studies have often combined the two phenomena. This may account for the significantly lower incidence of delirium in the year after DLB diagnosis compared to the year prior to diagnosis, as the phenomenon is likely to be recognised by clinicians as a core clinical feature of DLB, especially if the delirium were to present without psychosis [19]. Two of the three participants whose delirium episode was induced by international air flights in this study experienced transient speech abnormalities with the disturbed level of consciousness and did not report psychosis, unlike the remaining cohort. Further research into the link between delirium and cognitive fluctuations may shed light on overlapping pathophysiological mechanisms.

By assessing delirium relative to the appearance of other core and supportive features of the disease, our study explores the temporal trajectory of the progression of the disease in such patients. In this study, caregivers of participants identified cognitive change and parkinsonism as the earliest presenting symptoms of subsequent DLB. This differs from the study by Auning et al. [23], where the second most common presenting symptom was visual hallucinations. This difference may reflect the acquisition of the cohort from a movement disorder clinic, whose patients are likely to present with more motor features. Two-thirds of those experiencing delirium had multiple core and supportive features at the time of the delirium, with parkinsonism and apathy present in 44% of the participants. Three participants experienced delirium without additional clinical features of DLB at the time of delirium episode. The current study looks to inform clinicians and expand their suspicion for delirium-onset DLB from the acute cognitive decline post-delirium phenotype and thus improve diagnostic accuracy. With earlier suspicion for the subsequent development of DLB, it is hoped that clinicians will better avoid prescribing antipsychotic and anticholinergic medications in the prodromal period, reducing morbidity and potential mortality [10].

A limitation of this study is its retrospective nature, which relies upon caregiver recount, introducing a recall bias. This was reduced by correlating the caregiver account with information in the first clinical letter, as documented by the treating neurologist. Validated tools for caregiver identification of delirium episodes in non-hospitalised patients, years after their occurrence are lacking. This study demonstrated delirium episodes up to 12 years prior to diagnosis, and although attempts were made to adapt the DSM-V criteria to allow caregiver identification of delirium, and increase sensitivity, the study is limited by missing data bias and has potential for false positives. Given the retrospective nature and caregiver identification of delirium, we did not attempt to differentiate delirium episodes from cognitive fluctuations, given the shared phenomenology between these entities. To maximise sensitivity, all caregiver reported changes in arousal were considered potential delirium episodes. The authors acknowledge the potential for misclassification of cognitive fluctuations; however this was deemed justified given the exploratory nature of the study. We would assert that episodes with a clear inciting event and those featuring psychosis would more confidently be identified as delirium rather than cognitive fluctuation. Prospective studies assessing episodes, including delirium biomarkers, and contemporaneous validated scores for cognitive fluctuations and delirium, would help elucidate if these are indeed different entities or if both lie on a spectrum of the same pathophysiological process. The authors acknowledge the potential for selection bias; however, review of demographics showed similar age, sex and MoCA results between participatory and non-participatory cohorts. It is also clear that the small number of participants did not allow for a more comprehensive statistical analysis. However, the number of participants included here is comparable with a limited number of previously published studies [14, 19, 22–25].

Further research is required to inform the establishment of diagnostic criteria for prodromal DLB. In the meantime, we would propose that clinicians should suspect delirium-onset DLB in patients aged over 60, presenting with delirium especially triggered by international (long-haul) air travel. Clinicians should broaden their suspicion beyond the acute cognitive decline post-delirium phenotype, as they risk missing the majority of delirium-onset DLB cases. Until diagnostic criteria are developed and biomarkers evaluated in the delirium-onset DLB group, clinicians can stratify risk by assessing available biomarkers as outlined for DLB diagnosis. With an anticipated increase in access to α-synuclein based biomarker [41–43], further risk stratification should be conducted prospectively across centres. The present study raises suspicion that delirium-onset DLB has a more rapid progression to dementia. Thus, clinicians should make more rigorous efforts towards delirium prevention (pre-operative assessment, travel advice), which may in fact represent a potential disease-modifying strategy that should not be limited to those patients presenting with multiple delirium episodes.

## Supplementary Information

Below is the link to the electronic supplementary material.

Supplementary file1 (DOCX 14 KB)

## Data Availability

Data can be made available by the authors upon reasonable request.
