# Supplementary material for: Prevalence, timing and characteristics of delirium preceding dementia with Lewy bodies: a retrospective case series
Source: J Neurol. 2025 Jun 21;272(7):471. doi: 10.1007/s00415-025-13193-y (PMC12182519; doi:10.1007/s00415-025-13193-y)
Supplement: Supplementary file 1 — Supplementary file1 (DOCX 14 KB) [file 415_2025_13193_MOESM1_ESM.docx]

**Supplementary Table 1: Presenting feature as noted by caregivers in the prodromal period.**

|  | Entire cohort (n=34) | Prodromal delirium (n=9) | No delirium in prodromal period (n=25) |
| --- | --- | --- | --- |
| Core Feature present % (n) | n=14 | n=3 | n=11 |
| Visual Hallucinations | 0% (0) | 0% (0) | 0% (0) |
| Cognitive Fluctuations | 0% (0) | 0% (0) | 0% (0) |
| Parkinsonism | 32% (11) | 22% (2) | 36% (9) |
| RBD | 9% (3) | 11% (1) | 8% (2) |
|  |  |  |  |
| First prodromal feature % (n) | n=25 | n=8 | n=17 |
| Delirium | 6% (2) | 22% (2) | 0% (0) |
| MCI | 53% (8) | 56% (5) | 52% (13) |
| Psychiatric | 15% (5) | 11% (1) | 16% (4) |

Comparison of core features in the prodromal period. Values displayed as percentage (number). MCI, Mild cognitive impairment; RBD, Rapid eye movement sleep behaviour disorder.
